# Book Reviews

**Published:** 1908-11

**Authors:** 


### BOOK REVIEWS

THE READY-REFERENCE HANDBOOK OF DISEASES OF THE SKIN. By George Thomas Jackson, M. D., Chief of Clinic and Instructor in Dermatology, College of Physicians and Surgeons, New York. Sixth edition. 12mo., 737 pages, with 99 engravings and 4 plates, in colors, and monochrome. Cloth, $3.00, net. Lea & Febiger, Publishers, Philadelphia and New York, 1908.

Since the previous edition of this work, its author has been elected to the full Chair of Dermatology in the College of Physicians and Surgeons, of New York, a tribute both to the man and to his book. An examination of his pages affords some insight into the reasons for this appreciation. The most obvious characteristic is directness. The author clears the ground in his opening sections on Anatomy, Physiology, General Diagnosis and Therapeusis, and disposes of the moot subject of classification and nomenclature in the briefest and clearest may by means of a table, displaying the various diseases arranged in the most rational system, with the prominent primary lesion mentioned. The reader is now qualified to take up skin diseases in any order, and the most natural and practical is according to the alphabet. Herein lies the "Ready Reference" feature embodied in the title. Each disease is considered in full, beginning with synonyms and proceeding through the symptoms to the etiology, pathology and diagnoise, and to especially full sections on treatment covering all varieties and complications. The book is rich in formulas of proved value in this very trying class of cases. Answering the needs of students, as well as physicians, this work has merited the demand for six editions in sixteen years. It is well established in favor and repays it by frequent revisions, enabling its readers always to keep posted to date.

---

A TEXT-BOOK OF PHYSIOLOGY. For Students and Practitioners. By George V. N. Dearborn, A. M., (Harvard), Ph. D., M. D. (Columbia), Professor of Physiology in Tufts College, Medical and Dental Schools, Boston. Octavo, 550 pages, with 300 engravings and 8 colored plates. Cloth, $3.75 net. Lea & Febiger, Publishers, Philadephia and New York, 1908.

Professor Dearborn's new work enters its field, already rich in literature, well equipped to achieve a position in the forefront. It is easier to write verbosely than concisely, to state mony things rather than to determine what is really important. Our outhor conceives it to be the duty of a book to guide and instruct a reader presumably unacquainted with its subject beforehand. Instead of turning him loose in a mass of more or less arranged facts, a true text-book should present its principles and data in orderly, logical form, and should also indicate their mutual relations and bearings, so that the student mastering it may have a clear view of the whole subject. Dr. Dearborn obviously possesses both the requisite physiological knowledge and didactic ability, and his work, manifesting and combining these qualities, is certain to be appreciated by teachers as an excellent text-book for their students, and therefore as a first-class aid in the performance of their own duties.

---

PATHOGENIC MICRO-ORGANISMS, INCLUDING BACTERIA AND PROTOZOA. A Practical Manual for Students, Physicians and Health Officers. By William H. Park, M. D., Professor of Bacteriology and Hygiene in the University and Bellevue Hospital Medical College, New York. New (third) edition, thoroughly revised and much enlarged. Octavo, 648 pages, with 176 illustrations and 5 full-page plates. Cloth, $3.75, net. Lea & Febiger, Philadelphia and New York, 1908.

Dr. Park was the first to give concrete recognititon in book-form to the fact that diseases caused by animal organisms are almost as important to the human race as those resulting from low forms of vegetable life. It is true that the pathogenic bacteria, representing the vegetable kingdom, are more numerous than the disease-bearing protozoa, or animalcules, and it is also true that the latter are more difficult to cultivate and demonstrate, but no reason can justfy ignoring them. Professor Park, perceiving this deficiency, supplied it in the most effective manner by preparing chapters on the protozoa and placing them with others on bacteria in a single volume, where they could be studied together, both in similarity and contrast. His work was thus the first to cover all diseases caused by micro-organisms. The need for it and the acceptable way it supplies that need may be seen in the demand for three editions. In a subject of such intense activity, growth is very great, and accordingly the changes in this new edidion are extremely thorough-going. Like its predecessors, it is intended to answer the needs of the student and physicians to cover the whole subject of pathogenic micro-organisms from both standpoints.

GOLDEN RULES OF DIETETICS. The General Principles and Emperic Knowledge of Human Nutrition; Analytic Gables of Food Stuffs; Diet Lists and Rules for Infant Feeding and for Feeding in Various Diseases. By A. L. Benedict, A. M., M. D., Buffalo, N. Y. Published by C. V. Mosby, St. Louis. Price $3.00.

Benedict presents in this volume the general principles of the science and art of dietetics and gives succinct rules for the application of these principles. Being less dogmatic than "Golden Rules" on other subjects published by these publishers, we believe this book is an improvement and will be of distinct value to the physician who will bear in mind its precepts.

